# Serglycin is part of the secretory repertoire of LPS-activated monocytes

**DOI:** 10.1002/iid3.47

**Published:** 2015-02-11

**Authors:** Ingrid Benedicte M Kolseth, Trine Marita Reine, Tram Thu Vuong, Astri Jeanette Meen, Qiong Fan, Trond Geir Jenssen, Line Mariann Grønning-Wang, Svein Olav Kolset

**Affiliations:** 1 Department of Nutrition, Institute of Basic Medical Sciences, University of OsloOslo, Norway; 2 Department of Transplant Medicine, Section of Nephrology, Oslo University HospitalRikshospitalet, Oslo, Norway; 3 Metabolic and Renal Research Group, UiT The Arctic University of NorwayTromsø, Norway

**Keywords:** Cytokines, inflammation, lipopolysaccharide, monocytes, serglycin

## Abstract

Monocytes play multiple roles in the immune system, and are active in both acute and chronic diseases. Patients exposed to bacterial infections depend on monocytes in defense reactions, but excessive immune reactions may also cause morbidity through systemic inflammatory responses. Few studies have addressed the importance of proteoglycans, and in particular, the hematopoietic serglycin, in such monocyte immune reactions. Adherent primary monocytes were cultured in absence and presence of LPS. Media were analyzed by ELISA for detection of serglycin. Lysed cell fractions were used to determine the mRNA level of serglycin. Monocytes were also cultured on chamber slides to investigate if serglycin could be detected intracellularly by immunocytochemistry. Monocytes secreted serglycin, and LPS-stimulation increased the secretion. Secretion of inflammatory cytokines increased to a larger extent than serglycin. mRNA levels of serglycin were also increased, suggesting both increased expression and secretion. Immunocytochemistry revealed the presence of serglycin in intracellular vesicles, many destined for secretion. Serglycin containing vesicles increased in number and size when the cells were exposed to LPS. Intracellular vesicle localization and secretion of the proteoglycan serglycin is shown for the first time in primary human monocytes. Monocyte activation by LPS increased the expression and secretion of serglycin, suggesting roles for serglycin in inflammatory processes.

## Introduction

Non-resolving inflammation is a driving force in several acute and chronic diseases, and postulated to be one of the greatest contributors to the medical burden in developed countries.

Exaggerated or insufficient inflammatory responses from the innate immune system can lead to organ dysfunction and shock 1. Excessive inflammatory responses also cause morbidity and mortality in chronic diseases such as diabetes mellitus, atherosclerosis, and autoimmune diseases 2–4. Monocytes are potent innate immune cells, representing 2–10% of total leukocyte counts in circulating blood. They are important in immediate responses to infections, and the non-classical and the intermediate subpopulations are increased in numbers in several acute and chronic diseases 5. Pattern recognition receptors on the immune cells recognize pathogen-associated molecular patterns or damage-associated molecular patterns. Lipopolysaccharide (LPS) is a classic pathogen-associated molecular pattern which is found in the outer cell membrane of gram-negative bacteria. Monocytes are activated when LPS bind to Toll-like receptor 4 6. This activates a cascade of intracellular signaling pathways, culminating in increased production of cytokines such as tumor necrosis factor (TNF)α, interleukin (IL)-1α, IL-1β, IL-6, CXCL8, and transforming growth factor (TGF)-β 2,6.

Human blood monocytes are divided into three subsets; the classical strongly CD14-positive (CD14^++^CD16^−^) monocyte, the intermediate CD14- and CD16-positive monocyte (CD14^++^CD16^+^), and the non-classical monocyte, which is strongly CD16-positive (CD14^+^CD16^++^). The intermediate and the non-classical monocyte together are often referred to as CD16+ (CD16-positive) monocytes 7. By serum deprivation for 90 min, human peripheral blood mononuclear cells (PBMC) will adhere in vitro. Such adherent monocytes are dominated by CD16-positive cells 8. Levels of these cells are expanded in septic patients, in patients suffering from chronic kidney disease, during inflammatory diseases, and in patients with localized infections 9–12. These cells are potent producers of TNFα and IL-1β in response to LPS 8,13.

Monocytes also secrete proteoglycans (PGs) in response to LPS-stimulation 14. PG secretion in human monocytes is increased with various immunological stimuli, such as LPS 14. In early studies the secreted PGs were identified based on the nature of their glycosaminoglycan chains, not the protein core. Primary human monocytes express almost exclusively PGs with chondroitin 4-sulfate chains, whereas differentiated macrophages express both chondroitin 4-sulfate and chondroitin 4,6-disulfate (Chondroitin sulfate E) 15,14,16. Serglycin has been identified as the major PG in human monocyte cell lines 16,17, but has not been studied to any great extent in primary human monocytes. The major part is constitutively secreted under in vitro conditions and has an approximate molecular weight over 220 kDa 18. Cell fractions of primary human monocytes have previously been shown to contain more free chondroitin sulfate chains than intact PGs 16. In murine macrophages serglycin is also the major secreted PG 16,19, increased after LPS stimulation and important for the secretion of TNFα. Also, in endothelial cells LPS increase the secretion of serglycin 20. Serglycin has earlier been shown to co-localize with inflammatory mediators in secretory vesicles in endothelial cells, cytotoxic T-cells and mast cells, suggesting a role for serglycin in storage and secretion of inflammatory mediators 21,22. CD16-positive cells have been shown to be potent producers of classic pro-inflammatory mediators 10,13,23. The aim of this study was to investigate serglycin expression, localization, and secretion in cultured primary human CD16-positive monocytes 8, before and after LPS stimulation. Results presented suggest that serglycin expression and secretion is part of the inflammatory response in human monocytes and can be detected in intracellular vesicles.

## Materials and Methods

### Human monocyte culture

Peripheral blood mononuclear cells (PBMC) were obtained from Buffy coats suspended in endotoxin-free PBS (Sigma), using Lymphoprep density gradient (Axis-Shield Poc AS) according to the manufacturer's instruction. The PBMC fraction was suspended in RPMI1640 with L-glutamine (Gibco) and antibiotics (penicillin and streptomycin (Sigma)) and 5 mM of glucose (Sigma) without serum, and plated onto plastic chamber slides (Nalge Nunc International). Monocytes were allowed to adhere for 90 min at 37°C/5% CO_2_, before washing three times with RPMI1640 to remove non-adherent cells. The adherent cells were then cultured in RPMI1640, with L-glutamine/antibiotics, 10% heat inactivated fetal calf serum (Sigma), and 5 mM of glucose, and in presence or absence of 1 μg/ml LPS derived from *Escherichia coli* (O26:B26 from Sigma–Aldrich (Sigma)). The cultures were incubated for 3, 6 and 24 h, respectively. Adherent cells obtained by this method have previously been shown to be highly enriched in CD16-positive monocytes, as 60% of the cells in such cultures are CD16high. Only 20% of the cells were shown to be of the main blood monocyte population (CD14high)—also referred to as classical monocytes 8.

### Serglycin ELISA

Serglycin released from the adherent monocytes was measured in culture supernatants using an enzyme-linked immunosorbent assay (ELISA) according to Niemann et al. 24, using the Genesis software, on Thermo Electron Corporation Multiskan EX.

### Quantitative real-time PCR

RNA from primary monocytes was isolated using the E.Z.N.A total RNA kit 1 (Omega Bio-Tek). RNasin® Plus RNase Inhibitor (Promega) was added to the isolated RNA, and the RNA was stored at −80°C for later reverse transcription to cDNA. From each sample, 90 ng of total RNA was reverse transcribed using High Capacity RNA-to-cDNA Kit (Applied Biosystems). Quantitative real-time (qRT) PCR was performed with TaqMan Gene Expression Master Mix (Applied Biosystems), cDNA and dH_2_O up to 20 μl. Specific mRNA levels were determined by qRT-PCR performed on ABI PRISM 7900 HT Sequence detector system (Applied Biosystems), using TaqMan gene expression assays (catalogue no. Hs01004159_m1 for serglycin) and the housekeeping genes β-actin and RPL30 (catalogue no. Hs99999903_m1 and Hs00265497_m1, respectively; Applied Biosystems). All samples were run in triplicates, and the housekeeping genes and target genes were run on the same plate. The relative mRNA level for each transcript was calculated by the ΔΔcycle threshold (Ct) method 25.

### Immunocytochemistry

Adherent monocytes grown on chamber slides were washed three times in PBS, fixed in 4% paraformaldehyde for 10 min, and washed in PBS for 10 min. Both primary and secondary antibodies were diluted in 1.25% BSA containing 0.2% saponin and spun at 13000 rpm for 5–10 min at 4°C before use. The cells were incubated with primary antibody affinity purified rabbit anti-human serglycin (1 μg/ml, kindly provided by N. Borregaard) or the affinity purified concentration matched irrelevant control for serglycin (rabbit anti-Hemocyanin (KLH), Sigma–Aldrich), overnight at 4°C in a dark humidity chamber. Some wells were only incubated with dilution buffer without primary antibodies, to test for specificity of the secondary antibody. The slides were then washed twice for 5 min in PBS and incubated with a secondary antibody, Alexa Fluor 488 conjugated goat anti-rabbit IgG (1:600, Invitrogen), for 90 min in a dark humidity chamber at room temperature. Finally, the slides were washed in PBS for 10 min, washed in MQ-H_2_O, air dried, and mounted using SlowFade Gold antifade reagent with DAPI (Invitrogen). Cells were examined with an Olympus FluoView FV1000 confocal microscope (Olympus Corporation, Tokyo, Japan) and a PlanApo 60x/1.40 oil objective. All images were obtained by sequential scanning under the same setting using FV10-ASW 3.1 software. Image processing was performed with Photoshop CS4 software (Adobe).

### Statistical analysis

**Figure 1:**
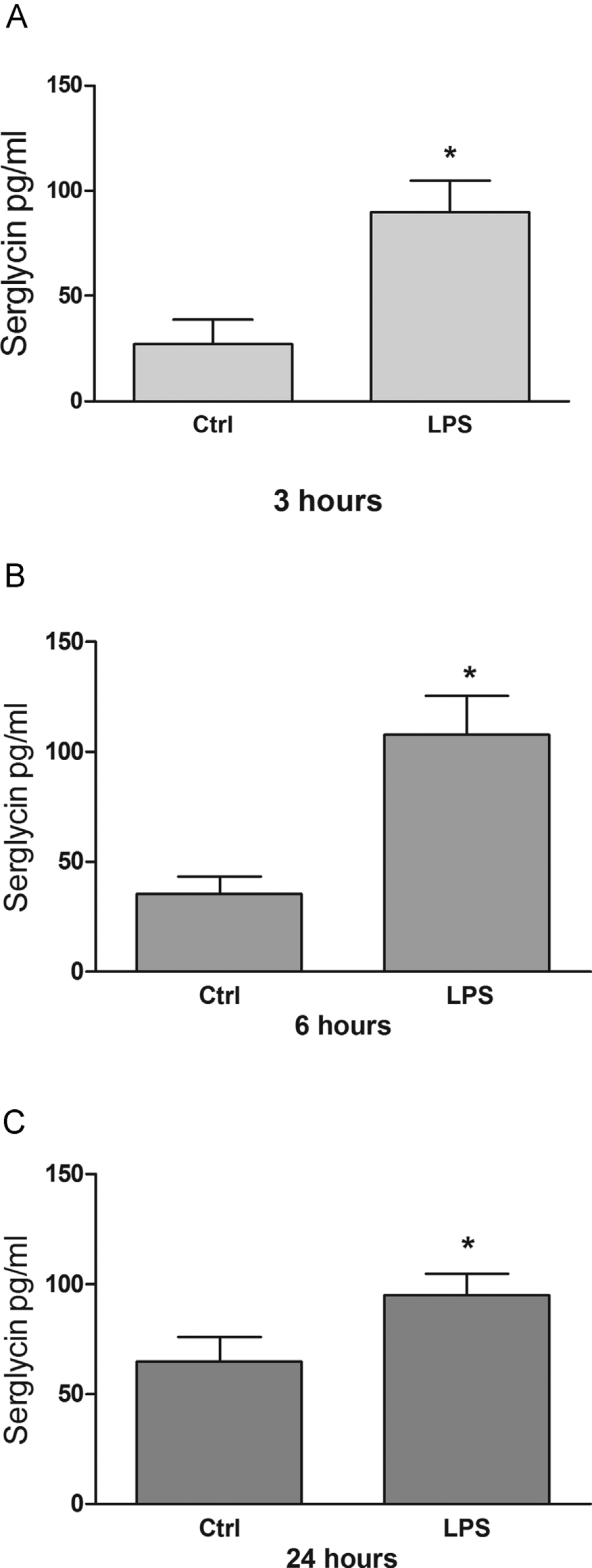
Secretion of serglycin in LPS-activated human adherent monocyte cultures is increased. Cultures of adherent human monocytes from seven separate donors were exposed to either LPS (1 μg/ml) or saline for 3 (A), 6 (B), or (C) 24 h, at 37°C with 5% CO_2_. Secreted serglycin found in the media was quantified by ELISA. *P ≤ 0.05.

**Figure 2:**
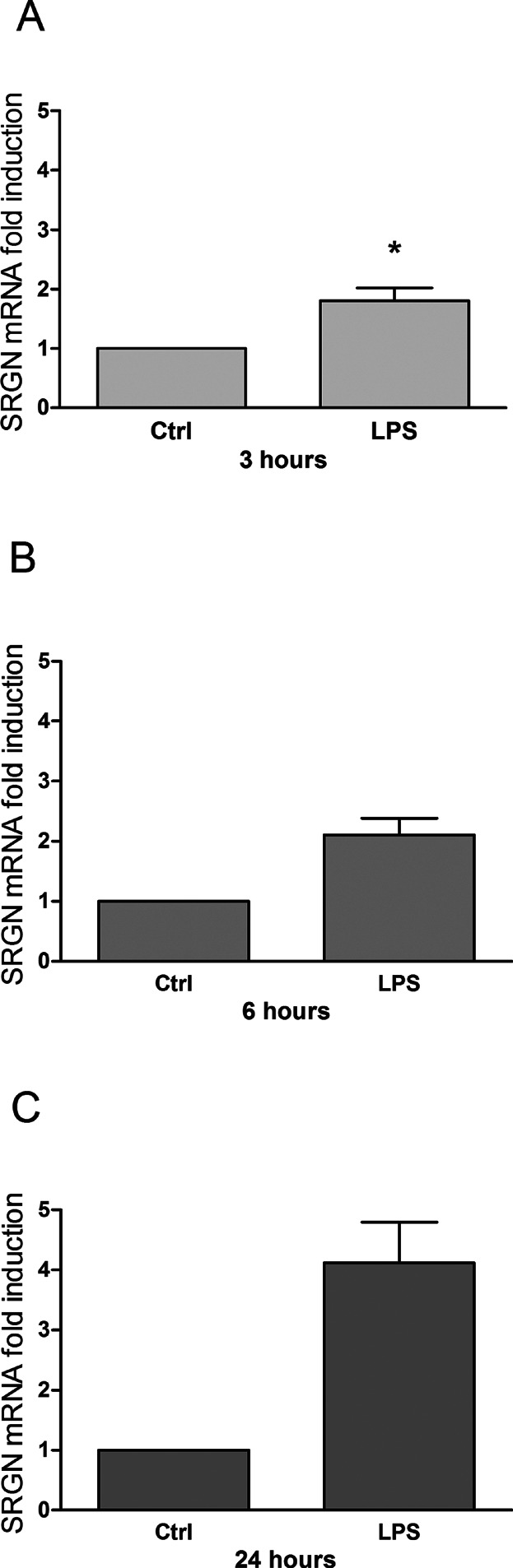
Serglycin mRNA expression in LPS-activated human primary monocytes is increased. Adherent monocytes from 4–6 healthy persons were exposed to LPS (1 μg/ml) or saline for 3 (A), 6 (B), and 24 (C) hours, at 37°C with 5% CO_2_. RNA was isolated and assayed for serglycin by quantitative RT-PCR. Data are shown as mean fold change from monocytes exposed to saline. *P ≤ 0.05.

**Figure 3:**
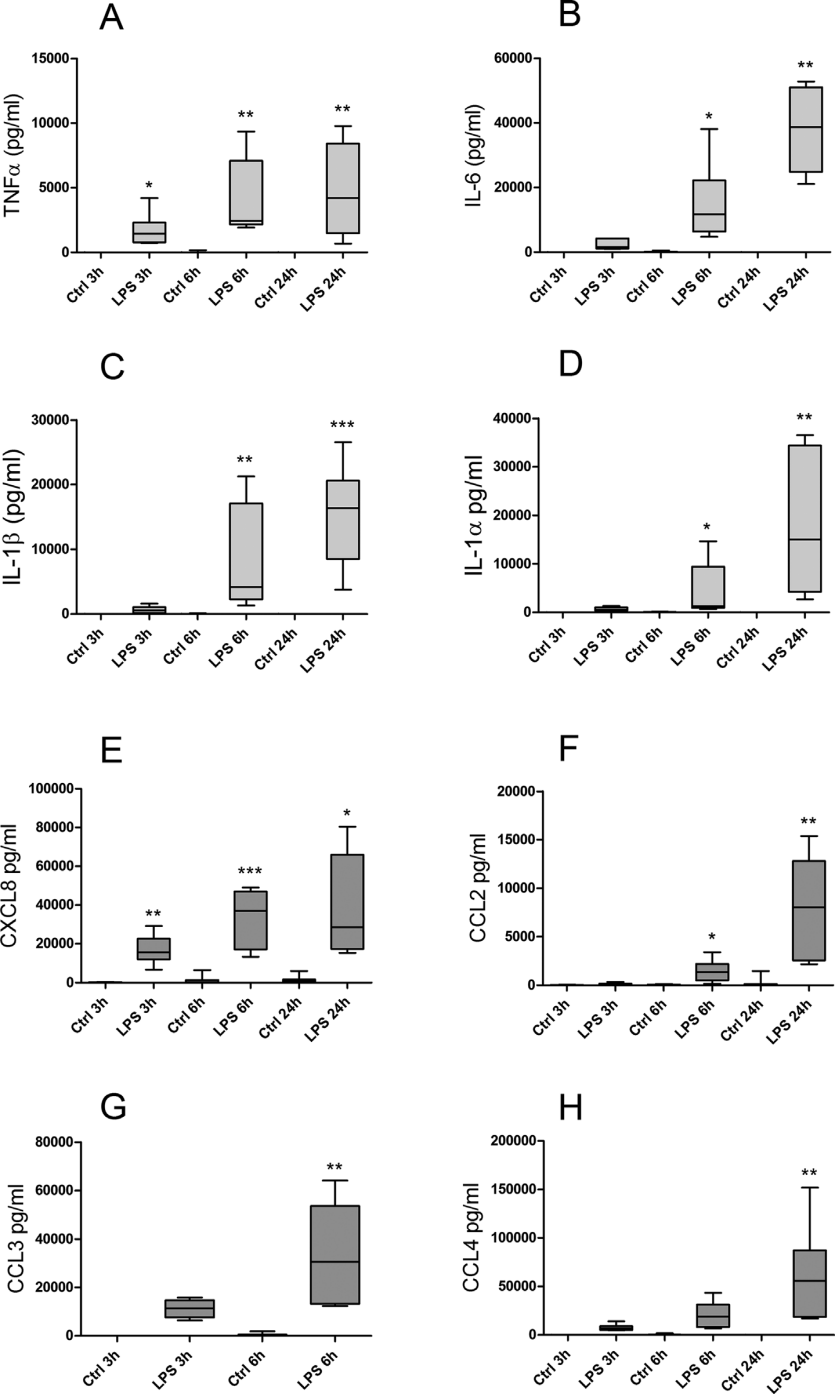
LPS-induced cytokine and chemokine release. Human adherent monocytes from seven separate donors were treated with saline or LPS (1 μg/ml). After 3, 6, and 24 h of incubation at 37°C with 5% CO_2_, media were harvested and analyzed for TNFα (A), IL-1α (D), IL-1β (C), IL-6 (B), CCL2 (F), CCL3 (G), CCL4 (H), and CXCL8 (E) with a multiplex antibody bead kit. Levels of CCL3 from LPS-stimulated monocytes at 24 h were above range in most donors at this time-point (hence, data not shown). Each LPS stimulation point is compared to its own control. *P ≤ 0.05, **P ≤ 0.005, ***P ≤ 0.001.

Data in Figures 1 and 2 were analyzed with GraphPad Prism 5.03. Differences between the groups were analyzed using the non-parametrical Wilcoxon matched pairs test. Differences of *P* ≤ 0.05 were considered significant. Differences between the groups in Figure 3 were analyzed using the non-parametric Friedman test for repeated measures, with multiple corrections performed with the Dunn's post-hoc test with columns to be compared selected on the basis of experimental design.

## Results and Discussion

### LPS increased the secretion and expression of serglycin in monocytes

To investigate the possible implications of serglycin in monocytes exposed to LPS stimulation, supernatants were subjected to ELISA using an antibody against human serglycin. The results obtained show that serglycin is a secretory product of LPS-activated human primary monocytes. The PG serglycin has previously been shown to be a secretory product of the monocyte-like cell lines U937 16 and THP-1 cells 17. In this study serglycin secretion in primary human monocytes is demonstrated for the first time.

In monocytes incubated with LPS, secretion of serglycin was significantly increased 3.3 times after 3 h, as can be seen in Figure 1A. After 6 h the increase was 3.0 times, whereas the stimulatory effects observed after 24 h incubation were weaker (approximately 1.5 times)—but still significant. In addition, the increased secretion of serglycin is supported by a corresponding increased mRNA expression (Fig. 2), evident by the doubling of mRNA levels after 3 and 6 h and approximately fourfold increase after 24 h compared to controls. In unstimulated cells the increase in serglycin secretion is time dependent and lower than in LPS stimulated cells. The effect of LPS on serglycin is rapid and secretion is sustained at this high level through the entire incubation period. It is worth noting that the high serglycin secretion is followed by a higher level of serglycin mRNA in stimulated compared to control cells after 24 h, suggesting feedback mechanisms in the LPS treated cells.

In control cells there is a constitutive and low secretion of serglycin, while the secretion of chemokines and cytokines is at a very low level and not increasing with time. The effect of LPS on the secretion of chemokines and cytokines was determined by a multiplex antibody bead kit. LPS was a potent inducer of secretion of TNF-α, IL-1α and β, CXCL8, CCL2, 3 and 4 and IL-6, as can be seen in Figure 3. Stimulatory effects were obvious after 3 h and increased in a time dependent manner. These results, demonstrating increased secretion of chemokines and cytokines, were further supported by the large increase also seen in mRNA levels, determined by qRT-PCR (data not shown) for all these cytokines and chemokines, thereby confirming effects of LPS on both expression and secretion. Accordingly, the secretion pattern for serglycin differed from those of the chemokines and cytokines investigated, suggesting an early role of serglycin in inflammatory reactions. From these results we conclude that serglycin may be regarded as a part of the secretory repertoire of human monocytes, and thereby the innate immune defense. The increase in secretion after LPS stimulation suggests that serglycin has functions related to inflammatory reactions, possibly involving serglycin in secretion and transport of inflammatory mediators.

Early studies on PG secretion in monocyte cell lines did not allow for identification of serglycin, but was based on radiolabeling of GAG-chains with ^35^S-sulfate. Activation with LPS increased ^35^S-PG secretion twofold, whereas interferon-γ treatment attenuated the secretion 14, demonstrating that PG secretion in human monocytes is subject to different types of regulation depending on type of stimuli.

The increased secretion of inflammatory mediators from LPS-activated monocytes after stimulation coincides with an increase in serglycin secretion. This may reflect the importance of serglycin as a secretory vehicle—and thereby dampening or promoting effects on inflammation. This suggests that serglycin may be an important factor in monocyte defense mechanisms. We demonstrate that immediate secretion more than expression of serglycin, which is a later effect, is altered by an inflammatory stimulus.

In a study by Zernichow et al, TNFα secretion from macrophages derived from serglycin^−/−^ mice increased when stimulated with LPS. The increased level of TNFα was due to increased secretion, not synthesis 19. In the same study, secretion of MMP-9, IL-1α, and CCL3 was not altered 19. A study performed on LPS-activated endothelial cells showed that secretion of CXCL8 was unaltered by loss of serglycin 20. It is therefore possible that serglycin selectively interact with some chemokines more than others and that such interactions may differ between cell types.

### Serglycin is present in intracellular vesicles in monocytes

**Figure 4:**
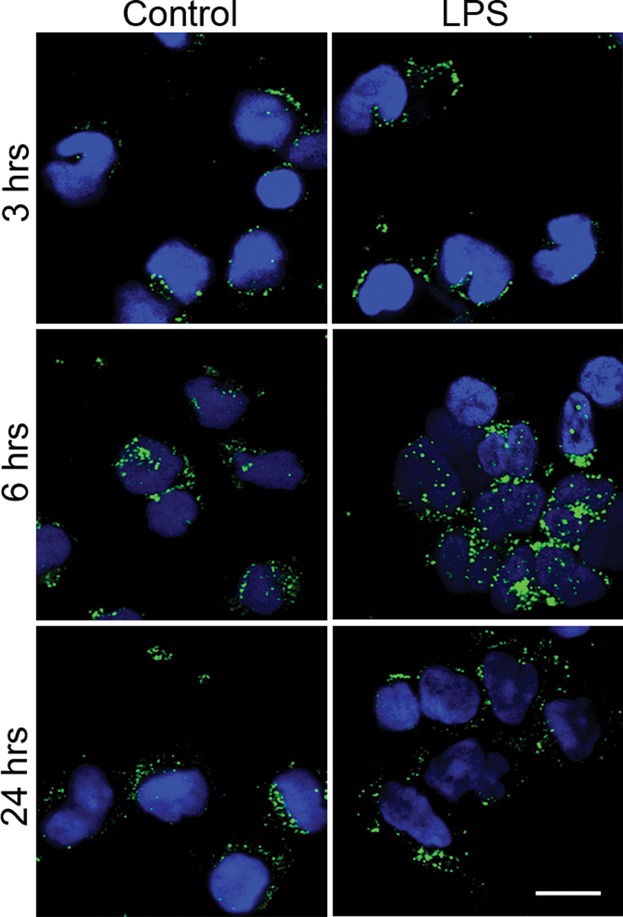
Immunostaining reveals intracellular vesicles containing serglycin in adherent human monocytes. The panels show adherent monocytes incubated in the presence (right) or absence (left) of LPS (1 μg/ml), for 3, 6, and 24 h. The cells were fixed, and stained for serglycin (green). Positive DAPI staining (blue) show the nucleus. Bar = 50 μM.

Serglycin secretion has not previously been studied in primary human monocytes in any great detail. To further investigate serglycin secretion in LPS activated monocytes, cells were fixed and subjected to immunocytochemistry. From Figure 4, it can be demonstrated that monocytes contained both large and small serglycin-positive vesicles at all three time points (3 h, 16 h, and 24 h—both in the presence and the absence of LPS. After 3 h of LPS stimulation, no significant differences in the distribution or sizes of the vesicles were observed. After 6 h however, LPS-stimulated cells showed stronger serglycin staining with an increased number of larger vesicles, compared to the unstimulated cells. In control cells, the vesicles were concentrated more closely to the plasma membrane. In LPS-stimulated monocytes serglycin containing vesicles tended to be more evenly distributed throughout the entire cytoplasm. After 24 h, however, the size and intracellular localization of serglycin positive vesicles were similar in control and LPS stimulated cells. The data presented on serglycin secretion using ELISA suggest that levels secreted after 24 h are still higher than in control cells, but at the same levels as after 3 h of stimulation. The lack of difference in serglycin staining after 24 h can, however, indicate that secretion reaches a peak after LPS stimulation and that the cells thereafter return to a state of basal level secretion, as in controls.

The immunocytochemical demonstration of serglycin in secretory vesicles, increasing in number and size after LPS stimulation, further supports the hypothesis that serglycin has functions related to the inflammatory response. One such function could be to regulate secretion of cytokines and chemokines, as has been demonstrated in other serglycin expressing hematopoietic cells 21 and endothelial cells 22.

### The extracellular role(s) of serglycin

Another intriguing aspect of serglycin secretion is the possibility that it may have functions in the extracellular environment of activated monocytes *following* secretion. It has been demonstrated that perforin and granzyme B delivered from cytotoxic T-cells to target cells are found in complex with serglycin, suggesting functions for serglycin-related targeted extracellular transport of inflammatory mediators 26. Moreover, in serglycin overexpressing cells it has been demonstrated that secretion of active proteases depend on the expression of serglycin 27. In monocytes, secreted ^35^S-PGs rich in serglycin was purified and added back to fresh cultures of monocytes. The ^35^S-PGs were not degraded to any great extent and remained in the culture medium. No cellular uptake or degradation could be demonstrated 28. We have also demonstrated that CCL3 binds to serglycin 29 and that deletion of the GAG binding domain in CCL3 reduced its chemoattractant activity 30. These data and the data presented here suggest that serglycin secreted from human monocytes has extracellular and inflammatory functions. An increased secretion of serglycin from human monocytes after LPS exposure suggests that serglycin can be involved in secretion, protection, and delivery of such partner molecules during inflammatory reactions.

### Future aspects

It has been suggested that serglycin may participate in the formation of chemotactic gradients of secretory products from different types of hematopoietic cells. Serglycin has also been detected on cells surfaces 31,32, including breast cancer cells, suggesting interactions with cell surface components 33. These are fascinating new aspect of PG biology 34,35 that deserves to be addressed in future studies. Moreover, serglycin may be secreted directly to the extracellular environment. It cannot be excluded that serglycin is retained within secretory vesicles or exosomes. Preliminary studies in our laboratory show the presence of serglycin in microparticles (Reine, unpublished data).

Possible interactions between intracellular serglycin and storage and secretion of inflammatory mediators should be studied in order to determine if serglycin has mainly proinflammatory or antiinflammatory abilities—or both. In order to explore the role of serglycin in immunological reactions, serglycin^−/−^ mice represent a useful in vivo model. Furthermore, secreted vesicles containing serglycin should be analyzed for possible complexes between inflammatory mediators and serglycin.

## Concluding Remarks

The data presented show that primary CD16-positive monocytes from healthy donors secrete serglycin as part of their inflammatory response to LPS. We also demonstrate for the first time that serglycin is found in secretory vesicles in primary monocytes. Together, these results suggest a role for serglycin in the innate immune defense system.
